# Complete chloroplast genome of a wild-type *Gardenia jasminoides* ellis (rubiaceae) adapted to island climate

**DOI:** 10.1080/23802359.2020.1861997

**Published:** 2021-02-03

**Authors:** Min Zhang, Wan-Dong Chen, Yuan-Yuan Li, Cheng Zhang, Zi-Han Chai, Yong-Fu Li, Shang-Wei Xie, Shao-Yong Deng, Yi-Fan Duan, Xian-Rong Wang

**Affiliations:** a Co-Innovation Center for Sustainable Forestry in Southern China, College of Biology and the Environment, Nanjing Forestry University, Jiangsu, Nanjing, China; b Nanji Islands National Marine Natural Reserve Administration, Zhejiang, Pingyang, China; c Jiangxi Academy of Forestry, Jiangxi, Nanchang, China

**Keywords:** *Gardenia jasminoides*, chloroplast genome, phylogenetic analyses

## Abstract

*Gardenia jasminoides* Ellis is a traditional aromatic and medicinal plant in China. Here, the complete chloroplast genome of a wild-type gardenia adapted to island climate was assembled. The assembled genome was 155,247 bp in length, with four typical regions, i.e., a large single-copy (LSC) region (85,414 bp), a small single-copy (SSC) region (18,235 bp) and two inverted repeats (IRs) regions (25,799 bp each). In total, 138 genes were predicted, including 90 protein-coding genes, 40 tRNA genes and eight rRNA genes. The overall GC content of the chloroplast genome was 37.5%. The chloroplast genome would provide more information for the phylogeography and phylogeny study of *G. jasminoides*.

*Gardenia jasminoides* Ellis, belonging to the family Rubiaceae, is a traditional aromatic and medicinal plant. The main officinal part of *G. jasminoides* is its dried ripe fruit. Chemical composition analysis finds that there are multiple secondary metabolites in the fruit of *G. jasminoides*, like iridoid glycosides, crocin and crocetin, which have broad spectrum anti-inflammatory properties and even anti-cancer effect (Qin et al. 2013; Chen et al. 2015, 2017). All these make *G. jasminoides* an important resource plant, and relative studies are arising. Zhao and Zhou (2020) reported the complete chloroplast genome of wild-type gardenia based on the material collected from Quanzhou, Fujian province, China. However, *G. jasminoides* is widely distributed in the area south of the Yangtze River, which is adapted to divergent habitats and may form different genotypes. In this study, the complete chloroplast genome of another wild-type gardenia adapted to island climate was assembled, which could provide more information for the phylogeography and phylogeny study of this species.

The material used in this study was collected from Nanji islands, Wenzhou, Zhejiang province of China (121°5′44.75″ E, 27°27′21.58″ N). The voucher specimen was kept in the herbarium of Nanjing Forestry University (accession number: NF2019102301). Fresh leaves of *G. jasminoides* were harvested and frozen in liquid nitrogen, then transferred to −80 °C refrigerator, immediately. Genetic DNA was extracted from these leaves for sequencing library construction. The library was then paired-end sequenced on the Illumina NovaSeq 6000 platform (Illumina Inc., San Diego, CA, USA) by Genepioneer Biotechnologies Co., Ltd. (Nanjing, China) with the standard Illumina re-sequencing protocols. In total, 6 Gb clean reads were obtained. After reads quality filtration, SPAdes 3.11.0 (Bankevich et al. 2012) and SSPACE (Boetzer et al. 2011) were alternately used for data assembly. Then, GapFiller v1.11 (Boetzer and Pirovano 2012) was further applied for gap filling. The complete sequence was primarily annotated by PGA (Qu et al. 2019) combined with GeSeq (https://chlorobox.mpimp-golm.mpg.de/geseq.html). Finally, IRscope (https://irscope.shinyapps.io/irapp/), an online tool was used to identify the borders of IR, LSC and SSC regions. The chloroplast genome of *G. jasminoides* assembled in this study was 1,55,247 bp in length with four typical regions. The lengths for LSC, SSC and IRs regions were 85,414 bp, 18,235 bp and 25,799 bp, respectively. So, it is longer than that reported by Zhao and Zhou (2020) for two insertion mutations at non-coding regions. The whole LSC region of *G. jasminoides* in Zhao and Zhou’s (2020) study was inverted, but inversion mutation was not detected in this work. Excluding these mutations, the identity between the two genomes is 99.99%. Finally, 138 genes were predicted in our study, including 90 protein-coding genes (81 species), 40 tRNA genes (32 species) and 8 rRNA genes (4 species). The overall GC content of the chloroplast genome was 37.5%.

Maximum likelihood (ML) tree was constructed to reveal the phylogenetic relationship of *G. jasminoides* with other species in Gentianales. Genome-wide alignment was conducted by MAFFT 7.158 (Katoh and Standley 2013). Then phylogenetic inference was performed in RAxML-VI-HPC (Stamatakis 2006) software under the GTR-gamma model. To assess the confidence of each internal node, rapid bootstrap method was applied with 1000 replications. The result showed that phylogenetic positions of all the taxa were successfully resolved (Figure 1). *Gardenia jasninoides* was placed in Rubiaceae clade and classed together with *Coffea arabica*, which indicated that *Gardenia* is more closely related to *Coffea* than other studied genera.

**Figure 1.**
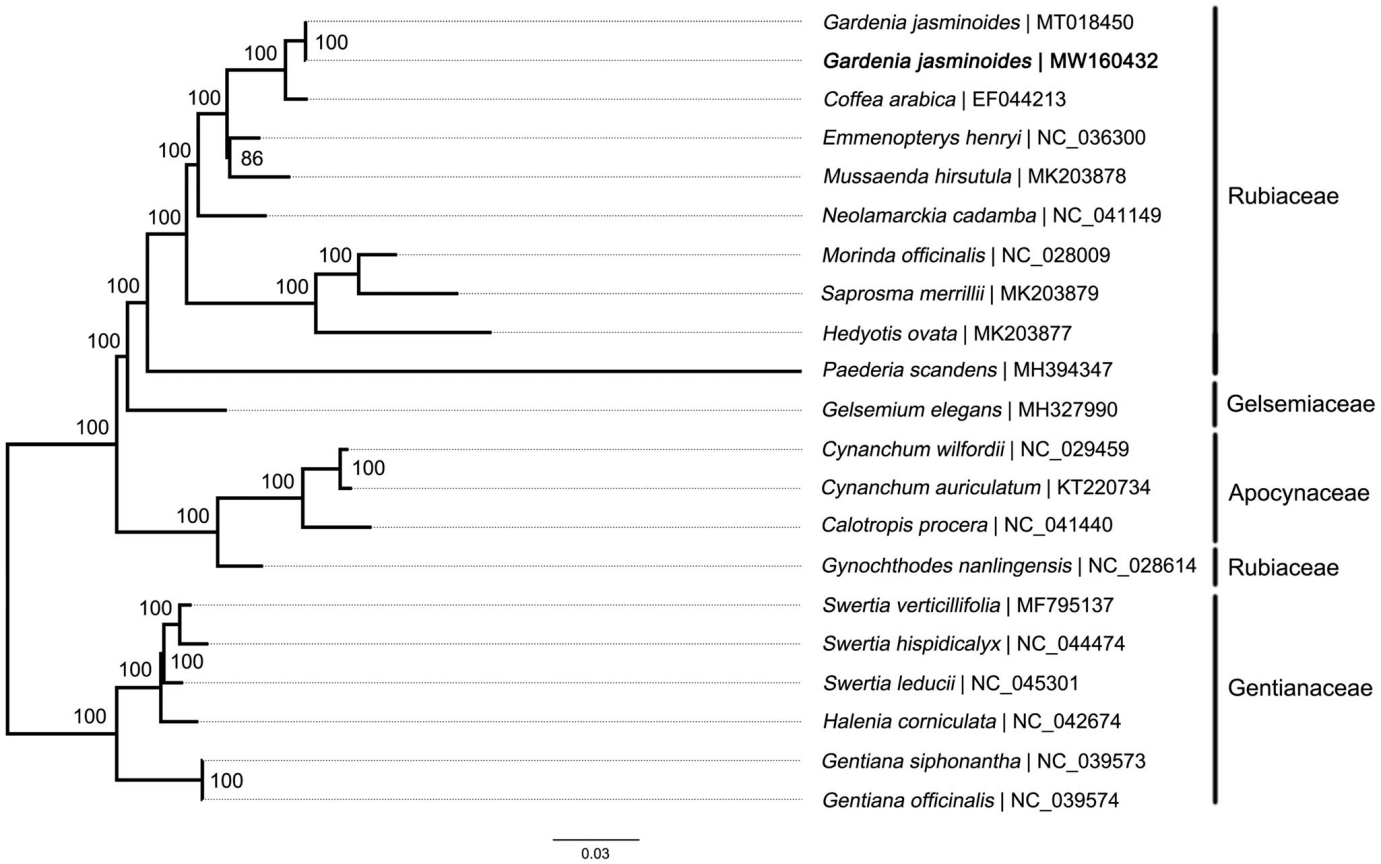
Maximum-likelihood tree based on the sequences of 21 chloroplast genomes from Gentianales. Numbers at tree nodes represent bootstrap values for 1000 replications. Number after ‘|’ shows the accession number in GenBank for each accession. The position of the wild-type *Gardenia jasminoides* reported in this study is marked in bold.

## Data Availability

The raw sequence data supporting this study are deposited in the National Center for Biotechnology Information Short Read Archive under BioProject ID PRJNA678106 (accession number SRP292439). The assembled genome and its annotation are openly available in GenBank of NCBI at https://www.ncbi.nlm.nih.gov, reference number MW160432.
